# The role of pre-treatment paraaortic surgical staging for cervical cancer in the EMBRACE criteria

**DOI:** 10.3332/ecancer.2022.1463

**Published:** 2022-11-03

**Authors:** Oscar Puga, Javier Retamales, Nicolás Saez, Miguel Urzúa, Miguel Saavedra, María Victoria Pérez, Dania Acuña, Karen García

**Affiliations:** 1 Unidad Oncología Ginecológica, Complejo Asistencial Sótero del Río, Av Concha y Toro 3459 - 8207257, Santiago, Chile; 2 Division of Obstetrics and Gynecology, School of Medicine, Pontificia Universidad Católica de Chile, Lira 40 - 8330023, Santiago, Chile; 3 Chilean Cooperative Group for Oncologic Research, Av Jose Manuel Infante 125 Of. 11 - 7500650, Santiago, Chile

**Keywords:** cervical cancer, laparoscopic lymphadenectomy, ovarian transposition, radiotherapy, EMBRACE

## Abstract

**Background:**

The State-of-the-Art Treatment for Locally Advanced Cervical Cancer (LACC) is Definite Radio-Chemotherapy based on the Image-guided intensity modulated External beam radiochemotherapy and MRI-based adaptive BRAchytherapy (EMBRACE) trial, according to the FIGO staging. This staging is based on clinical examination and imaging studies; however, there are limitations of imaging techniques which may result in adverse events or death due to insufficient or overtreatment. The aim of the study was to evaluate the feasibility and outcomes of surgical staging in LACC prior to radiotherapy (RT) to personalise target volumes for radiotherapy.

**Methods:**

From 2008 to 2018, 138 patients with FIGO 2018 stages IB3-IIIC2 cervical cancer underwent a pretherapeutic laparoscopic staging procedure. The pathological diagnosis was compared with the results of preoperative CT scan. Patients were treated with chemoradiotherapy tailored according to the staging results.

**Results:**

The mean patient age was 43 years, the mean body mass index was 27 kg/m^2^; most lesions were squamous cervical cancer (92%). Staging CT scan had a 77% concordance with the histological findings. Sensitivity was 29%, specificity 85%, positive predictive value 21% and negative predictive value 89%. Surgical staging led to change of stage in 24% of cases. Para -aortic dissection led to change the initially planned radiotherapy fields in 47% of the cases. Major complications included involuntary section of the inferior mesenteric artery (IMA) without clinical repercussion, an infected retroperitoneal haematoma and a symptomatic lymphocele requiring laparoscopic drainage.

**Conclusion:**

Laparoscopic staging before primary chemoradiation in patients with LACC was feasible, safe and reproducible, allowing reduction of the radiotherapy treatment volumes of patients.

## Highlights

- Lymph node status is considered the most important independent prognostic factor in Locally Advanced Cervical Cancer.
- Fédération Internationale de Gynécologie et d’Obstétrique (FIGO) 2018 does not define criteria to discriminate between malignancy and inflammation/infection of lymph nodes.
- Laparoscopic surgical staging is a safe procedure that led to a change in the clinical stage in 24% of patients.
- Laparoscopic surgical staging led to reduction of radiotherapy treatment volumes in 47% of patients.

## Introduction

Radiotherapy with cisplatin-based chemotherapy is the standard of care for locally advanced cervical cancer (LACC) patients. For such patients, the lymph node status is a treatment and prognosis determinant. In fact, local or distant nodal metastasis is considered the most important independent factor, potentially changing the planned treatment [1–3]. Until recently, the FIGO staging system for cervical cancer was primarily clinical, but imaging studies have been included since 2018 [4] into the staging, taking into consideration not only the tumour size, but also the lymph node involvement.

Both NCCN and ESTRO support offering radiotherapy treatment volumes based on the data generated by the EMBRACE group, which are currently standardised in the EMBRACE 2 clinical trial [5] and involve adding the para-aortic (PAo) region as treatment volume when involved lymph node is suspected in PAo or common iliac territory, or, when three or more pelvic lymph nodes compromised are suspected; however, treatment of PAo involves more toxicities than pelvic-exclusive treatment [6, 7].

Moreover, the choice of imaging for staging and radiotherapy planning is based on the availability of the technology and expertise. Most cervical cancers are diagnosed in low-resource settings where options such as modern cross-sectional and functional imaging (e.g. CT, MRI, positron emission tomography-computed tomography (PET/CT)) are either constrained or not accessible at all [8].

Surgical staging is considered part of clinical staging [9] as histological verification of tumour extension correlates better with the biological behaviour of the disease [10, 11]. Previously , laparotomy surgical staging was attempted, but this presented as a drawback large abdominal incision, with considerable peri and postoperative morbidity and delaying the start of radiotherapy [1, 6, 7, 10, 12–14].

As an alternative to laparotomy, laparoscopic surgery has proven to be a safe procedure, with low morbidity, allowing a shorter recovery time and, therefore, a quick return to work without delaying the start of radiotherapy [15–19]. Laparoscopic surgical staging can change the initially planned treatment for patients with LACC in 15%–43% of cases, considering the extension of the volumes to be irradiated. All patients with aortic lymph node metastases who do not receive treatment are dead within 3 years compared to those treated with radiotherapy who achieve survival rates of 20%–35% after 5 years [20–22].

The aim of our study is to evaluate the feasibility and outcomes of laparoscopic surgical staging in our hospital comparing the results with CT scan, providing a tailored treatment for each patient minimising over- and under-treatment.

### Objectives

- To describe the procedure.
- To describe the cohort in terms of demographics.
- To describe procedural findings during surgery.
- To describe ovarian transposition and transposed ovary as site of recurrence.
- To describe overall survival in the cohort by stage in terms of FIGO 2018.
- To describe overall survival in the cohort in terms of PAo lymph node status.
- To discuss clinical staging correlation with surgical staging.
- To discuss the change of radiotherapy treatment volumes due to histopathologic findings.

## Materials and methods

This was a prospective observational study conducted by the Gynecologic Oncology Unit of the Hospital Sótero del Río, designed to comply with STARD (Standards for Reporting of Diagnostic Accuracy Studies) guidelines [23]. The study was approved by the institutional review board. Informed consent was waived. Patients with primary, histologically confirmed, locally advanced (FIGO 2018 stages IB3 to IIIC2) invasive carcinoma of the cervix who had no contraindication to surgery were invited to participate and enrolled after consent. Patients were excluded from the study if they had undergone contraindications to laparoscopy, multiple medical malignancies, anticoagulant therapy, prior pelvic radiation therapy, prior pelvic or abdominal lymphadenectomy, and patients who had metastasis outside the pelvis. All patients were examined by at least three oncologists from the Gynecologic Oncology Unit. Central Radiology review was performed for all patients. Patients underwent extra-peritoneal or laparoscopic pelvic and abdominal lymphadenectomy within 2 weeks after CT. At the beginning of the surgery, we explored the entire abdominal cavity and peritoneal washing cytology was sampled. We excluded patients if they had intraperitoneal disease. Lymph node and peritoneal samples were evaluated and centralised by the chief pathologist. Data were collected in the electronical clinical record at baseline, surgery, first month after radiotherapy and at follow-up, every 3 months the first 2 years and every 6 months up to year 5. Patients were censored at 5 years of follow-up.

## Results

**Figure 1.**
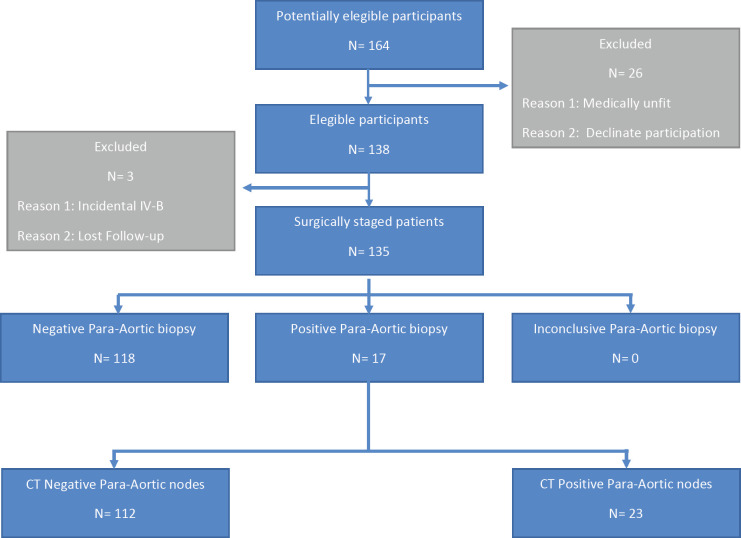
STARD flow diagram.

From 2006 to 2018, 138 patients were studied, of which 33 were operated transperitoneally and 105 extraperitoneally. The average age was 43 years (range: 20–71), ten patients were nulliparous, with average parity of 2.5 children (range: 0–9). The average total body mass index (BMI) was 27 (19–44). The STARD flow diagram is presented in Figure 1.

The most frequent histological type was squamous cell carcinoma in 127 patients (92%), 6 adenocarcinomas, 2 adenosquamous carcinomas, 2 undifferentiated carcinomas and 1 large cell carcinoma.

The mean surgical time was 127 minutes (45–200), in three patients the extraperitoneal route was changed to the transperitoneal route due to rupture of the peritoneum, not being able to achieve adequate pneumoperitoneum. There was no conversion to laparotomy. Twenty-nine ovarian transpositions were performed. The average estimated blood loss was 55 mL (0–300), and the average hospital discharge time was at 46 hours (16–144). There was no need for transfusions. As far as intraoperative complications are concerned, there was an involuntary section of the inferior mesenteric artery without clinical repercussion. In the postoperative period, one patient presented an infected retroperitoneal haematoma, treated medically but with long hospitalisation (144 hours). Another patient presented a symptomatic lymphocele requiring laparoscopic drainage.

Peritoneal cytology was positive only in one patient, who had evident tumour involvement in the cervical serosa. There was no involvement of other organs or intra-abdominal spread in the remaining patients.

The average number of resected PAo lymph nodes was 13 (3–31), and in 17 (12.4%) patients, there was histologically confirmed metastasis.

In 26 patients with pelvic lymphadenopathy greater than 2 cm, selective lymphadenectomy was performed as debulking. In 18 of these patients, there was lymph node metastasis, and 6 of these patients also had PAo metastasis. In the remaining eight patients with negative pelvic nodes for metastasis, all PAo nodes were negative for metastasis.

The cohort is presented in Table 1.

To evaluate the change of behaviour in the volumes of radiotherapy treatment, a consensus was reached with two Radiation Oncologists participating in the committee based on EMBRACE 2 criteria, reviewing the radiological reports and images of the 138 patients. Consensus was reached in 128 of the 138 cases, with 10 cases that could not be evaluated. Of the 71 patients who had an indication for extended RT, only 14 (11%) were histologically positive and 57 (45%) were negative. Of the 57 patients who did not have an indication for extended RT, only 3 (2%) were histologically positive and 54 (42%) were negative. Of the 128 evaluable patients, 45% would have received extended-field radiotherapy without needing it and 2% would not have received it if they needed it, which in total represents a change of behaviour in 47% of the cases.

**Figure 2.**
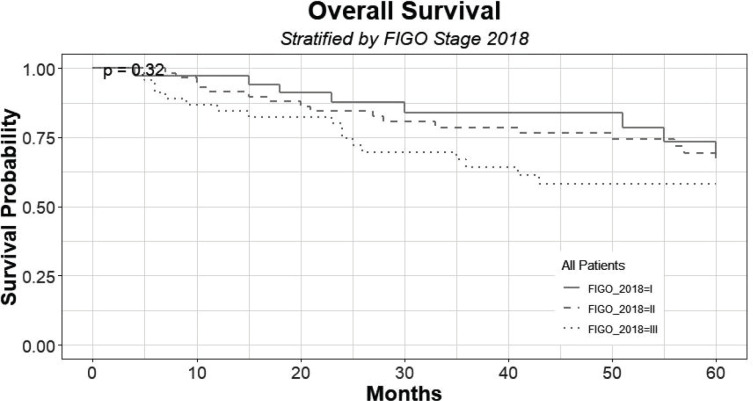
Survival distribution of patients at 60 months of follow-up, according to FIGO 2018, grouped by stage I, II and III.

In the follow-up period, there was no tumour recurrence in transposed ovaries. Figure 2 shows the survival distribution of the patients at 60 months of follow-up, according to the FIGO 2018 staging; including the censored patients that can be seen across the curves. The 60-month survival of patients with stage I, II and III was 67%, 66% and 58%, respectively, with no statistical differences (*p* = 0.65).

**Figure 3.**
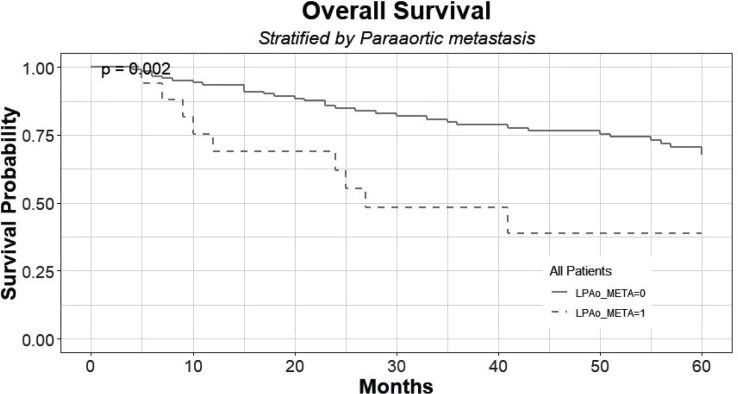
Overall survival of patients with histologically confirmed PAo metastases (LPAo_META = 1) compared to patients with no PAo metastases (LPAo_META = 0).

The 5-year overall survival of patients with histologically confirmed PAo metastasis was 38% (C.I: 0.20–0.95) compared to 67% (C.I: 0.59–90.5) of patients with no PAo metastasis (*p* < 0.05) (Figure 3). There are seven patients alive that are censored for not completing the long-term follow-up yet.

Staging CT had a 76.9% concordance with the histological findings. The sensitivity (95% confidence interval (95% CI)) of the method was 0.29 (0.10, 0.56), specificity (95% CI) 0.84 (0.76, 0.90), positive predictive value (95% CI) 0.21 (0.07, 0.42), negative predictive value (95% CI) 0.89 (0.82, 0.94). PAo dissection led to upstaging in 9.7% of the patients (confirmed PAo metastasis in non-suspicious imaging patients) and to downstaging in 14.2% (no PAo metastasis in imaging suspicious patients) of the patients.

Based on local imaging criteria, which are the same as EMBRACE 2 (PAo adenopathy, common iliac adenopathy, three or more pelvic adenopathies) [5], 55% of the patients had indication for PAo radiotherapy. PAo dissection led to a change in target volume of radiotherapy to exclusive pelvic volume in 52% of the cases. From the univariate analysis, the presence of histologically confirmed aortic metastasis was the main factor associated with a change in behaviour in relation to the volumes of radiotherapy *p* < 0.05.

## Discussion

Performing laparoscopic PAo lymphadenectomy as a staging of cervical cancer is a feasible, safe and efficient procedure, presenting low postoperative morbidity, little use of analgesics and opiates, allowing early discharge. It also allows the performance of ovarian transposition outside the irradiation field in the same surgical act, managing to preserve hormonal function in young women without increasing ovarian metastasis. The most important is that in 47% of patients, the laparoscopic staging changed the original treatment, avoiding PAo radiotherapy in 45% of the patients. PAo metastasis shows a worse prognosis.

The incidence of positive peritoneal cytology for squamous cell carcinoma is very low, as reported in the literature, and is probably not needed as part of the cervical cancer staging procedure [24].

We decided to perform lymph node dissection from aortic artery bifurcation to IMA due to the low prevalence of isolated metastases over this artery, which reaches only 3% according to Leblanc *et al* [25], reducing the potential additional morbidity in more extensive dissections.

It is important to notice that FIGO 2018 [4] does not define criteria to discriminate between malignancy and inflammation/infection on imaging, which is left to the physician’s discretion. It is the physician who must give an opinion on whether the case looks suspicious enough to upstage it or not. In general, 10 mm is considered the upper limit for normal nodes (short axis diameter) [26].

In 28 patients with suspicious pelvic adenopathies larger than 2 cm in diameter, we performed surgical resection of them. In 18 (70%) patients, we found metastasis, in the other 8 patients the nodes were negative, and, in these patients, metastasis was not found in the resected PAo nodes either. The debulking of pelvic lymph nodes bigger than 2 cm had no impact in overall survival, probably due to the low number of patients, but it has an impact on decreasing the radiation volumes and improving the prognosis assessment of that group.

Lymph node staging surgery prior to radio-chemotherapy can be cost effective even when PET-CT shows no evidence of metastasis [27]. Despite negative images, PAo metastasis can be present in up to 8% when performing surgical staging [28]. In our cohort, 12.4% of the patients had histological metastases in PAo surgical staging.

Adam *et al* [29] performed a systematic review of 12 studies with 778 patients and showed that the sensitivity of PET for PAo lymph node detection was only 40%. Ramirez *et al* [30] proved that with negative PET-CT for pelvic and PAo lymph nodes, there were 12% of positive PAo lymph nodes (12% false negatives) and this increased to 22% if the PET-CT was suspicious in the pelvis and was negative in the PAo territory. The landmark GOG 233, which also surgically staged a prospective cohort with PET-CT available, concluded that diagnostic CT had a sensitivity of 42% that changed to 50% after considering PET results, but this failed to demonstrate its superiority (*p* = 0.052); this is applicable to our cohort which obtained a sensitivity of 29%.

Regarding prognosis of the patients, Dabi *et al* [31] compared 377 patients with surgical staging versus 270 with clinical staging. After 38 months of follow-up, the surgical group presented a better prognosis in terms of odds ratio (OR), represented in disease-free survival (OR 0.64) and overall survival (OR 0.43). Gold *et al* [32] also showed benefits in progression-free survival (50% versus 36%) and overall survival (54% versus 40%) when comparing surgical versus clinical staging. Marnitz *et al* [33] found differences in disease-free and overall survival in stage IIB in terms of hazard ratio (HR) (HR 0.51) when comparing both groups, but their results can be due to insufficient power.

Considering toxicities, in the prophylactic PAo irradiation and pelvic radiotherapy groups in Radiation Therapy Oncology Group (RTOG) 79–20, the incidences of 10-year grade 4–5 toxicities from radiotherapy were 8% and 4% (*p* = 0.06), respectively, and the mortality rates due to radiotherapy complications were 2% and 1% (*p* = 0.24), respectively [7]. In the trial of the EORTC radiotherapy group, the 4-year grade 3–4 digestive complications were 8.0% and 3.5% (*p* = 0.005) in the prophylactic PAo irradiation and pelvic radiotherapy groups, respectively [6]. So, although measuring toxicity was not an objective of the trial, in our cohort PAo dissection led to a change in target volume of radiotherapy to exclusive pelvic volume in 45% of the cases, which represents evidence of personalised oncology.

## Conclusion

Although PET-CT is considered the best imaging method to detect lymph node involvement, its sensitivity is only 40% for detection of lumboaortic mestastasis [29, 30, 34], plus, Gallach *et al* [35] analysed the IAEA Medical imAGIng and Nuclear mEdicine global resources database and concluded that at least 96 countries should upscale their PET-CT services and more than 200 additional PET-CT scanners would be required to fulfil their needs [35]. It is precisely in Latin America in general and in low- and middle-income countries where there is a greater deficit of diagnostic imaging methods, countries where the incidence of cervical uterine cancer is still high, with screening programmes that have not been efficient, where we face diagnoses in advanced stages and high mortality. Therefore, there are a low percentage of patients who have access to PET-CT, performing clinical staging with CT. On the other hand, although Intensity Modulated Radiation Therapy (IMRT) has partly reported a decrease in the toxicity of radiotherapy [35], it is not readily available in our countries [36]. Given these limitations and our own results, it is even more justified to perform laparoscopic surgical staging in patients with LACC and thus be able to offer our patients better medicine with individualised therapies, decreasing the volume of radiotherapy and reducing its adverse effects in the long term.

## Authors’ contributions

Oscar Puga: Conceptualisation, methodology, reviewing and supervision; Javier Retamales: Data curation, software, reviewing; Nicolás Saez: Investigation, editing and original draft preparation; Miguel Urzúa: Investigation and editing; Miguel Saavedra: Investigation; María Victoria Pérez: Investigation; Dania Acuña: Investigation; Karen García: Investigation.

## Conflicts of interest statement

The authors have nothing to disclose in relation to this manuscript.

## Funding

The authors have no financial support to declare.

## Figures and Tables

**Table 1.** Main characteristics of the patients.

*N*	138
Age years, (range)	43 (20–71)
Parity, average (range)	2.5 (0–9)
BMI, average (range)	27 (19–44)
Histology	
Squamous cell carcinoma	127
Adenocarcinoma	6
Adenosquamous carcinoma	2
Undifferentiated carcinoma	2
Large cell carcinoma	1
Surgical time, min, average (range)	127 (45–200)
EBL (mL)	55 (0–300)
Hospital discharge, hours (range)	46 (16–144)
PAo lymphnodes average (range)	13 (3–31)
PAo metastases *N*	17 (12.4%)
FIGO stage	
IB3	22 (16%)
IIA2	1 (1%)
IIB	32 (23%)
IIIA	1 (1%)
IIIB	16 (12%)
IIIC1	44 (32%)
IIIC2	22 (16%)

EBL: estimated blood loss
